# Early-onset status epilepticus in patients with acute encephalitis

**DOI:** 10.1097/MD.0000000000004092

**Published:** 2016-07-29

**Authors:** Romain Sonneville, Eric Mariotte, Mathilde Neuville, Sébastien Minaud, Eric Magalhaes, Stéphane Ruckly, Marie Cantier, Guillaume Voiriot, Aguila Radjou, Roland Smonig, Jean-François Soubirou, Bruno Mourvillier, Lila Bouadma, Michel Wolff, Jean-François Timsit

**Affiliations:** Univ Paris Diderot, Sorbonne Paris Cité, Assistance Publique–Hôpitaux de Paris, Hôpital Bichat–Claude-Bernard, Service de Réanimation Médicale et des Maladies Infectieuses, rue Henri-Huchard, Paris Cedex, France.

**Keywords:** encephalitis, outcome, status epilepticus

## Abstract

Status epilepticus (SE) is a common complication of acute encephalitis, but its determinants and prognostic value in this setting are not known.

Risk factors for early-onset SE (within 48 hours of intensive care unit [ICU] admission) in consecutive adult patients with all-cause encephalitis admitted to the medical ICU of a university hospital (1991–2013) were evaluated by multivariate logistic regression analysis. To examine the prognostic value of SE, patients were classified into 3 groups: no SE, nonrefractory SE (NRSE), and refractory SE (RSE). Poor neurologic outcome was defined by a modified Rankin score of 4 to 6.

Among the 290 patients, 58 (20%, 95% CI: 15%–25%) developed early-onset SE, comprising 44 patients with NRSE and 14 patients with RSE. Coma (adjusted odds ratio [OR]: 3.1, 95% CI: 1.5–6.3), cortical lesions on neuroimaging (adjusted OR: 3.7, 95% CI: 1.8–7.8), and nonneurologic organ failure(s) (adjusted OR: 13.6, 95% CI: 4.9–37.7) were found to be independent risk factors for SE. By contrast, a bacterial etiology had a protective effect (adjusted OR: 0.3, 95% CI: 0.1–0.7). Age, body temperature, and blood sodium levels were not independently associated with SE. Poor neurologic outcomes were observed at day 90 in respectively 23% (95% CI: 17%–28%), 23% (95% CI: 10%–35%), and 71% (95% CI: 48%–95%) of no SE, NRSE, and RSE patients (*P* < 0.01). After adjusting for confounders, RSE, but not NRSE, remained independently associated with 90-day mortality (adjusted OR: 6.0, 95% CI: 1.5–23.3).

Coma, cortical involvement on neuroimaging, and nonneurologic organ failure(s) are independent risk factors for SE in patients with acute encephalitis. Conversely, a bacterial etiology is associated with a lower risk of SE.

These findings may help identify patients who may benefit from prophylactic antiepileptic drugs.

## Abstract 2

Supplemental Digital Content is available in the text

## 1 Introduction

Acute encephalitis is a severe neurologic condition caused by inflammation of the brain parenchyma, usually due to an infectious or immune-mediated process.^[1]^ Mortality rates among adult patients range between 7% and 18% and neurological sequelae are reported in more than 50% of survivors.^[2–4]^ Patients with acute encephalitis may require intensive care unit [ICU] admission for various reasons, including impaired consciousness, respiratory failure, or complicated seizures.^[2,4]^

Seizures occur in 32% to 36% of adult patients with encephalitis and are a potentially important cause of neurological deterioration.^[3,4]^ They are influenced by several factors, including the patient's history, central nervous system (CNS) inflammation, and cortical lesions.^[5,6]^ The risk of seizures may also differ depending on the cause of encephalitis, being reported in more than 40% of patients with encephalitis of viral origin.^[5]^ The prevalence of seizures seem to be even higher in patients with immune-mediated forms such as anti-NMDA receptor (NMDAR) encephalitis (up to 70%).^[7]^

Status epilepticus (SE) is reported in 5% to 18% of adults with acute encephalitis and has a major impact on treatment decisions and neurologic outcomes.^[2,3]^ Although seizure therapy may be an important element in the treatment of acute encephalitis, data on the possible beneficial effect of seizure prophylaxis are lacking.^[8]^ Recently, encephalitis was described as a rare cause of SE, associated with younger age and higher refractoriness.^[9]^ Identification of patients at a high risk of seizures and SE could help with the design of future trials of primary and secondary seizure prophylaxis in acute encephalitis. It is unclear whether SE contributes to poor neurologic outcomes or simply reflects the extent of brain injury in the most severe cases of encephalitis.^[10]^ A previous study suggested that SE was associated with an increased risk of in-hospital mortality,^[2]^ but the prognostic significance of seizures and SE in adults with encephalitis is controversial. Indeed, more recent studies showed no significant association between seizures or SE and 3-month neurologic outcomes.^[3,4]^ Recently, acute encephalitis was found to be associated with the onset of super-refractory SE,^[11]^ a condition carrying a very poor prognosis.^[12]^ In a previous study of patients undergoing continuous electroencephalographic monitoring, electrographic seizures occurred in 33% of cases and were independently associated with poorer outcomes.^[13]^ Moreover, in a recent study, the presence of seizures and SE at the onset of encephalitis was independently associated with an increased risk of postencephalitic epilepsy in survivors.^[14]^ The aim of the present study was to identify risk factors for SE in a large cohort of patients with all-cause encephalitis and to examine the impact of SE on 3-month vital and neurologic outcome. 

**Box 1:**
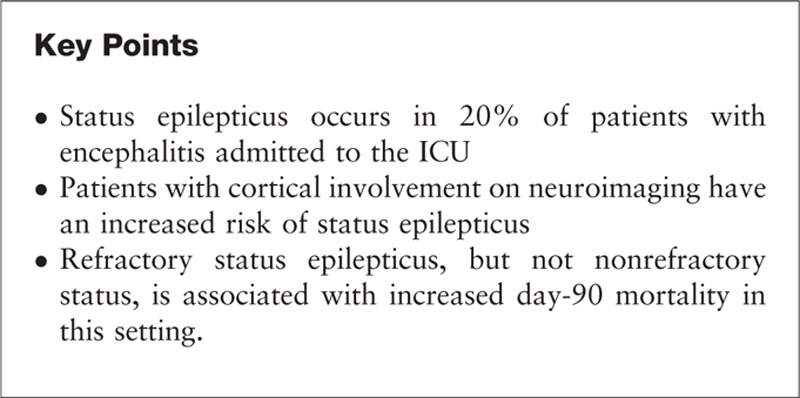
no caption available

## 2 Methods

### 2.1 Patients

All consecutive adults with a diagnosis of probable or definite encephalitis admitted to the medical ICU of Bichat–Claude-Bernard University Hospital, Paris, France, between 1 January 1991 and 31 December 2013 were included. Potential cases were identified from the hospital's computerized medical record system using ICD-10 codes for encephalitis. Patients were included if they fulfilled the following diagnostic criteria for acute encephalitis before or at ICU admission^[15]^: encephalopathy (altered consciousness that persisted for more than 24 hours, with lethargy, irritability, or a change in personality and behavior), and 2 or more of the following: fever or history of fever (≥38.0 °C) during the presenting illness; seizures; focal neurological abnormalities; cerebrospinal fluid (CSF) pleocytosis (more than 4 white blood cells per microliter); abnormal electroencephalogram (EEG) findings, and neuroimaging suggestive of encephalitis. The exclusion criteria were: alternative acute CNS disease; missing data on day 90 outcome; absence of encephalitic sign; *Streptococcus pneumoniae* or *Neisseria meningitidis* meningitis with secondary encephalitic features; isolated brain abscess; and AIDS-defining CNS diseases.

### 2.2 Data collected

We recorded the patients’ history and clinical, laboratory, and brain neuroimaging findings at admission, including the following scores: Glasgow coma scale (GCS, or the last GCS score before sedation when relevant),^[16]^ prior health status as assessed with the Knaus score,^[17]^ and the simplified acute physiology score 2.^[18]^ Poor functional status was defined by a Knaus score of C or D. Patients were considered comatose if the GCS score was below 8.^[19]^ The immunocompromised patient category included patients with HIV infection/AIDS, patients on immunosuppressive therapy, and patients with solid or hematologic neoplasms. Nonneurologic organ failure was defined as the need for invasive mechanical ventilation, vasopressors, and/or renal replacement therapy within 48 hours of ICU admission. Aspiration pneumonia was considered to occur in patients at risk of oropharyngeal aspiration (witnessed aspiration event, markedly depressed consciousness, and/or swallowing disorders), and was diagnosed on the basis of the following criteria: a new radiographic infiltrate compatible with pneumonia, predominating in the right lower lobe; and symptoms or signs of lower respiratory tract infection.^[20,21]^

### 2.3 Encephalitis etiology

Patients presenting with a suspicion of encephalitis were screened for common causes of infectious encephalitis in Europe, including CSF polymerase chain reaction (PCR) for HSV-1, VZV, and enterovirus; *Mycobacterium tuberculosis* CSF PCR and cultures; India ink stain, fungal cultures, and CSF cryptococcal antigen. In immunocompromised patients, additional CSF PCR for CMV, EBV, HHV6, HIV, and JC virus were routinely performed. Other conditional studies were performed depending on host factors, geographic factors, and presence of specific signs suggestive of an immune-mediated cause. Infectious encephalitis was defined by positive PCR, serology, culture, or histopathology. Immune-mediated encephalitis was defined by the presence of antigen-specific antibodies in serum and/or CSF.

Patients were classified into 6 groups: Herpes simplex virus encephalitis; Varicella-zoster virus (VZV) encephalitis; Bacterial causes (i.e., *Mycobacterium tuberculosis*, *Listeria monocytogenes*, leptospirosis, *Mycoplasma pneumoniae*, and syphilis); other infectious causes; immune-mediated causes, including acute disseminated encephalomyelitis and anti-NMDAR antibody encephalitis; and undetermined etiology. These causes were grouped into 3 main categories: infectious, immune-mediated, and undetermined, as previously described.^[15]^ For statistical analysis, patients of the whole cohort were dichotomized into bacterial versus nonbacterial (viral, immune-mediated, and undetermined) causes of encephalitis.

### 2.4 Status epilepticus

We collected data on seizure events that occurred in the prehospital setting and after hospital admission. We defined SE as 5 minutes or more of: continuous clinical and/or electrographic seizure activity; or recurrent seizure activity without recovery (return to baseline) between seizures.^[22]^ Patients with SE were further characterized into those with general convulsive SE (GCSE, i.e., generalized tonic-clonic movements of the extremities, with mental impairment, with or without focal neurological deficits in the postictal period) and those with nonconvulsive SE (i.e., seizure activity on the EEG without clinical signs of GCSE). Patients were considered to have refractory SE (RSE) if they continued to have either clinical or electrographic seizures after receiving appropriate doses of a benzodiazepine followed by a 2nd acceptable antiepileptic drug (AED). Patients were considered to have nonrefractory SE (NRSE) if the seizures ceased after adequate doses of a benzodiazepine, with or without a 2nd-line AED.

### 2.5 EEG recordings

Intermittent EEG recording was performed within 24 hours of onset of suspected encephalitis or seizures in patients with persistently altered mental status. Recorded data included background activity, reactivity, the presence and degree of focal or diffuse slowing, and epileptic discharges. Focal sharp waves and spikes, periodic-lateralized epileptiform discharges and generalized periodic discharges were grouped together as focalization. The presence of burst suppression or electric silence was noted.

### 2.6 Outcomes

Neurological outcomes were evaluated by reviewing the medical charts and/or by contacting the physicians in charge of the patient, and were graded 90 days after ICU admission by using the modified Rankin scale. Poor neurologic outcome was defined by a modified Rankin score of 4 to 6.^[4]^ Patients discharged within 90 days with a disability had their charts reviewed and were classified according to the latest available data. Patients discharged from hospital within 90 days following ICU admission without disability were considered to have a good outcome.

### 2.7 Standard protocol approvals, registrations, and patient consent

The local ethics committee approved the study protocol. Informed consent was not required but the patients or relatives were informed of the study whenever possible.

### 2.8 Statistical analysis

Patient characteristics were described as counts (percent) and median (interquartile range) for qualitative and quantitative variables, respectively, and were compared between groups by using the Chi-square or Mann–Whitney test, as appropriate. Patient characteristics were described with missing data. Prior to logistic regression analysis, missing data were imputed as the median for quantitative variables and the mode for qualitative variables. Logistic regression models were used to identify independent predictors of SE. The factors included in the initial models were clinically relevant variables with *P* values < 0.10 in univariate analysis. The linearity of the logit of quantitative variables was assessed by using cubic spline functions. Clinically relevant 2-by-2 interactions were tested in the final model. Forward stepwise variable elimination was then performed. Final *P* values below 0.05 were considered significant. Discrimination was assessed by using the area under the receiver–operator characteristic curve and calibration by using the Hosmer–Lemeshow goodness-of-fit test. Independent predictors of 90-day mortality were assessed with the same method. The SE variable (no SE/NRSE/RSE) was forced in the final model. We performed internal validation by using a bootstrapping procedure, which was done by taking a large number of samples (1000 independent replicates) of the original one. This technique provides nearly unbiased estimates of the confidence intervals of the odds ratio (OR) of the independent covariates. Analyses were performed with SAS 9.4 software (SAS Institute Inc., NC).

## 3 Results

### 3.1 Patients

Of the 420 patients retrieved from the queries in the computerized medical record system, 130 were excluded (e-Figure 1). A total of 290 patients with a median age of 39 years were analyzed (interquartile range 29–57), of whom 177 (61%, 95% CI: 55%–67%) were male. Their baseline characteristics are shown in Table 1. Poor functional status before admission was recorded in 23 cases (8%, 95% CI: 5%–11%), and 47 patients (16%, 95% CI: 12%–20%) were immunocompromised. Sixty-three patients (22%, 95% CI: 17%–27%) were comatose on admission, and 145 patients (50%, 95% CI: 44%–56%) were febrile. Seizure events were documented in 99 (34%, 95% CI: 29%–40%) patients, including 41 patients with uncomplicated seizures and 58 patients who met criteria for SE. Data on the prehospital use of AEDs and sedation is presented in e-Table 1.

**Table 1:**
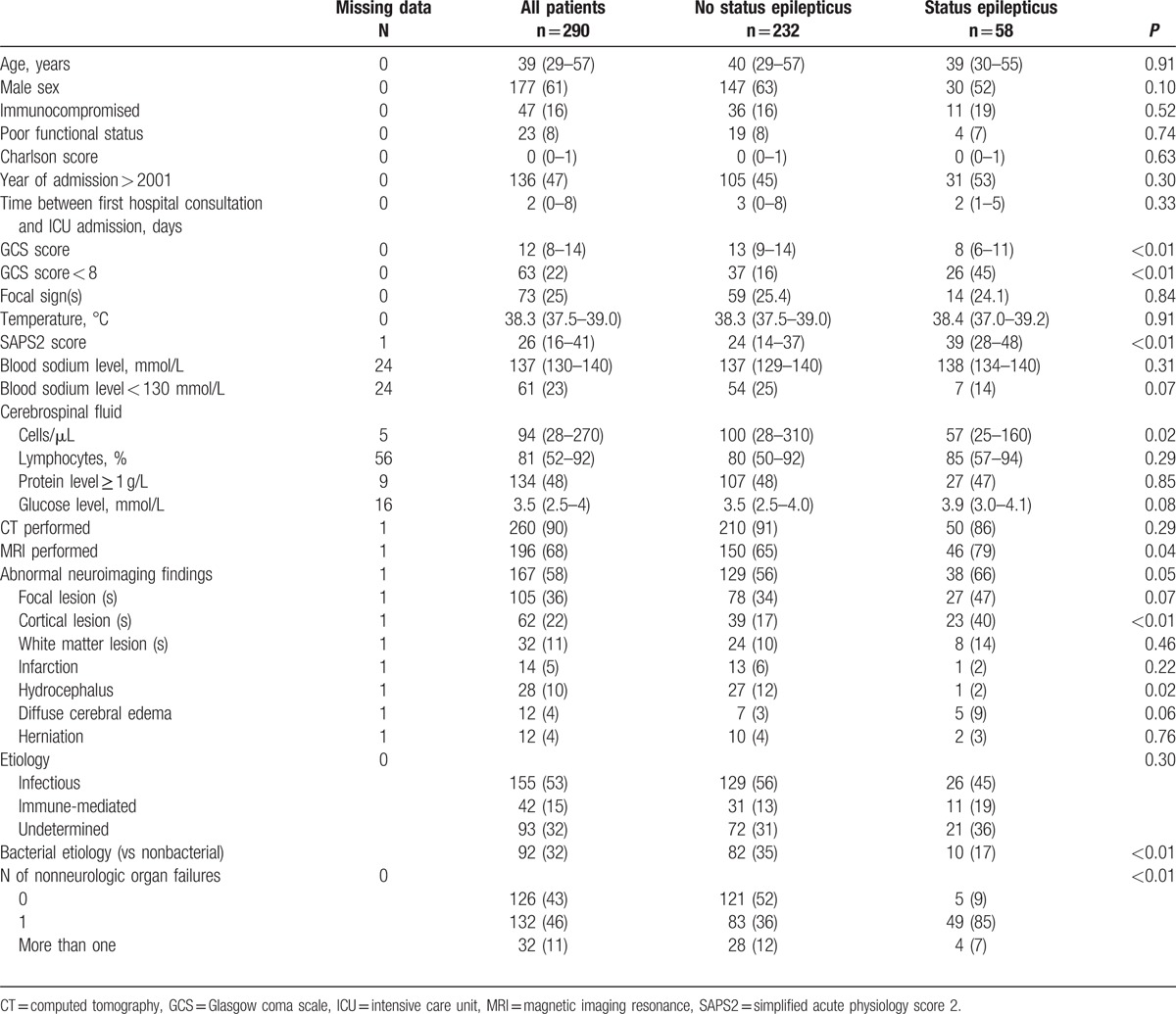
Baseline characteristics.

### 3.2 Patients with status epilepticus

**Figure 1:**
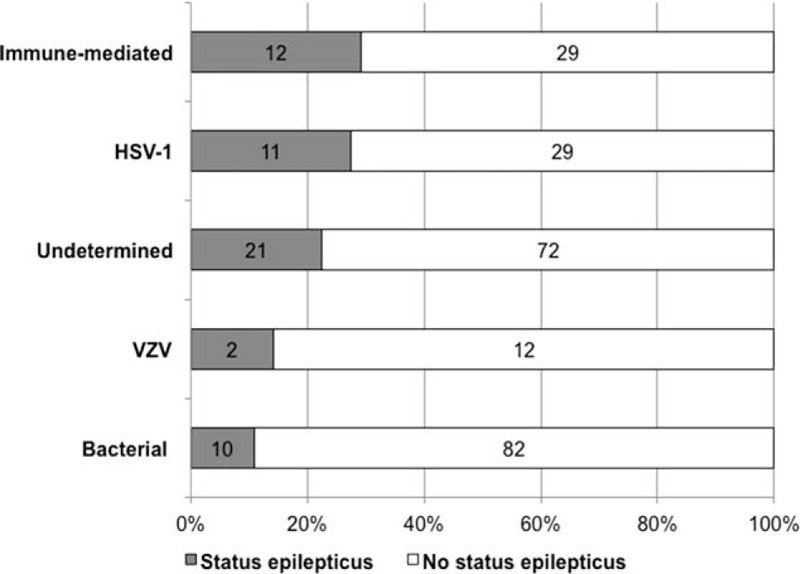
Cause-specific prevalence of status epilepticus in adults with encephalitis. HSV = Herpes simplex virus, VZV = Varicella-zoster virus.

SE occurred in 58 (20%, 95% CI: 15%–25%) patients within 48 hours of ICU admission (e-Figure 1), comprising 46 patients with convulsive SE and 12 with nonconvulsive SE. The time between hospital admission and SE onset was 2 (1–5) days. SE onset usually occurred before ICU admission, prehospital SE onset being observed in 21/58 cases (36%, 95% CI: 24%–49%), in-hospital but pre-ICU onset in 29/58 cases (50%, 95% CI: 37%–63%), and in-ICU onset in 8/58 (14%, 95% CI: 5%–23%) cases. The cause-specific prevalence of SE is shown in Fig. 1. The highest prevalence of SE was observed in patients with immune-mediated encephalitis (12/41, 29%, 95% CI: 15%–43%) or HSV-1 encephalitis (11/40, 28%, 95% CI: 14%–41%).

All 58 patients with SE received first-line i.v. benzodiazepine therapy (clonazepam or diazepam). Second line i.v. AEDs included phenobarbital (n = 24), phenytoin (n = 13), sodium valproate (n = 5), and levetiracetam (n = 2). There were 44 cases of NRSE and 14 cases of RSE. Compared to NRSE patients, RSE patients had higher Charlson scores and poorer functional status at ICU admission (e-Table 2). Patients who developed RSE received various 3rd-line intravenous AEDs, including high-dose midazolam (n = 10), propofol (n = 2), and pentothal (n = 6). Compared to NRSE, univariate analysis showed that RSE was significantly associated with higher mortality and poorer neurologic outcome at day 90 (e-Table 2).

### 3.3 EEG findings in patients with status epilepticus

Detailed EEG data were available for 51/58 (88%, 95% CI: 80%–96%) patients with SE. Generalized slowing was recorded in 44/51 cases (86%, 95% CI: 77%–96%), focalization in 27/51 (53%, 95% CI: 39%–67%), electric seizures in 18/51 (35%, 95% CI: 22%–48%), background activity in 5/51 (10%, 95% CI: 2%–18%), electric silence in 4/51 (8%, 95% CI: 1%–15%), and a burst suppression pattern in 1 (2%, 95% CI: 0%–6%).

### 3.4 Risk factors for status epilepticus

Compared to patients who did not develop SE, patients with SE had lower GCS scores and higher SAPS 2 scores, and were more likely to require invasive mechanical ventilation on ICU admission (Table 1). In univariate analysis, SE was significantly associated with lower CSF cell counts and with neuroimaging abnormalities. By contrast, SE was not associated with epidemiologic characteristics or comorbidities. Coma (adjusted OR: 3.1, 95% CI: 1.5–6.3), cortical involvement on neuroimaging (adjusted OR: 3.7, 95% CI: 1.8–7.8), and nonneurologic organ failure(s) (adjusted OR: 13.6, 95% CI: 4.9–37.7) (Table 2). By contrast, a bacterial etiology (versus nonbacterial etiology) had a protective effect (OR = 0.3, 95% CI 0.1–0.7).

**Table 2:**
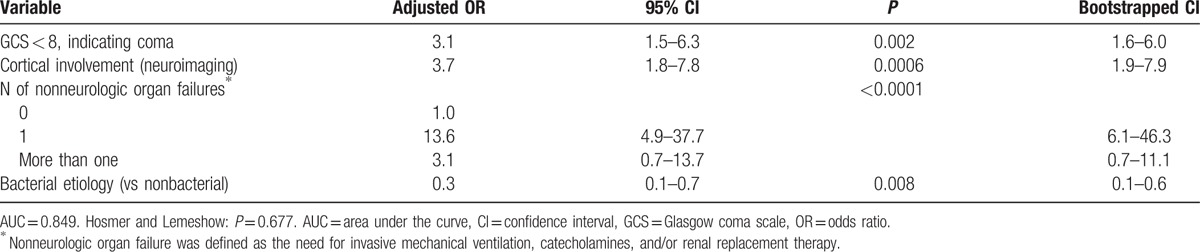
Risk factors for status epilepticus, multivariate analysis.

### 3.5 Outcome

**Figure 2:**
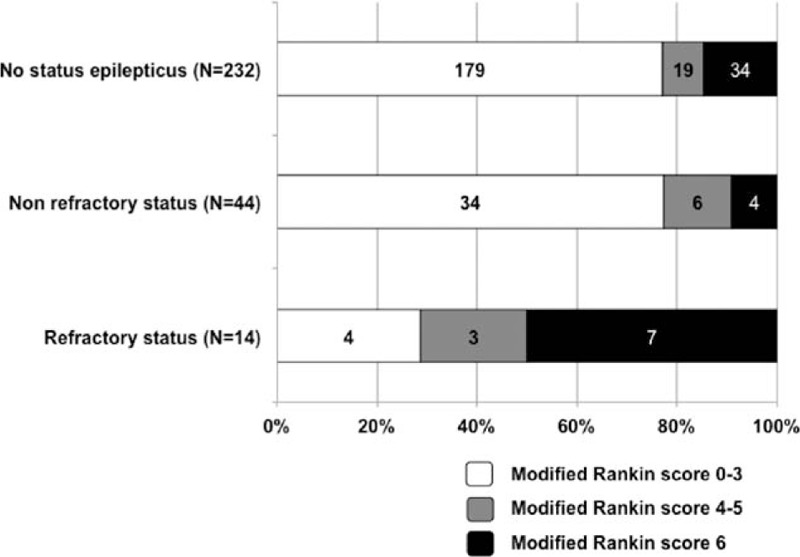
Day-90 neurologic outcomes.

The 90-day mortality rate was 16% (95% CI: 11%–20%) overall (45/290 patients), and was significantly higher in patients who developed RSE (e-Figure 1). Indicators of mortality were subjected to multivariate analyses. After adjusting for confounders, RSE, but not NRSE, remained independently associated with higher 90-day mortality (adjusted OR: 6.0, 95% CI 1.5–23.3) (Table 3). Patients with RSE had poor 90-day neurologic outcomes in 71% (95% CI: 48%–95%) of cases (Fig. 2).

**Table 3:**
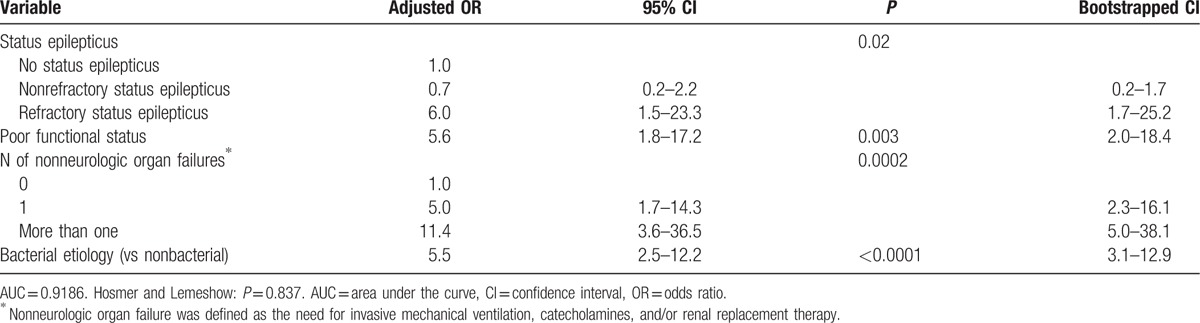
Predictors of day 90 mortality, multivariate analysis.

## 4 Discussion

In this analysis of a large cohort of adults with acute encephalitis followed for 3 months after initial ICU admission, early-onset SE occurred in 20% of cases. We identified 4 clinically relevant parameters independently associated with SE onset, namely coma on ICU admission, cortical involvement on neuroimaging, nonbacterial etiology, and nonneurologic organ failure(s).

The frequency of SE observed here (20%, 95% CI: 15%–25%) is slightly higher than previously reported,^[2,3]^ possibly because our cohort included more patients with severe encephalitis. Indeed, higher seizure rates have been reported in pediatric cohorts and in specific subgroups of patients with infectious encephalitis of viral origin^[5,6]^ or anti-NMDAR encephalitis.^[7]^ Cortical lesions on neuroimaging were a strong independent predictor of SE, confirming the findings of a previous study of 148 adults and children with encephalitis of viral origin.^[5]^ The strong independent association between SE onset and cortical involvement is in line with studies of stroke patients, in whom cortical involvement and hemorrhagic stroke were independently associated with an increased risk of symptomatic seizures.^[23]^ Contrary to a previous study, we found no association between age and SE, possibly because we only included adult patients and focused on SE and not all seizure types.^[5]^ The independent association between coma and SE observed here should be interpreted cautiously. On the one hand, altered mental status may be due to ongoing nonconvulsive seizures, a phenomenon increasingly recognized in ICU patients with unexplained coma.^[24]^ On the other hand, this association may simply reflect an incomplete return to baseline neurological status following repeated seizures, without ongoing seizure activity. In those patients, repeated spot EEG or continuous EEG monitoring is strongly recommended to rule out undetected seizure activity, especially when neuroimaging studies fail to explain their altered mental status.^[25]^ Interestingly, nonneurologic organ failure on ICU admission was independently associated with the risk of SE. This highlights the potentially complex interplay between systemic stress (e.g., hypoxia, severe sepsis, metabolic disturbances) and the risk of seizure activity. It also indicates that early and appropriate management of systemic complications might reduce the risk of seizure activity.

Encephalitis is devastating neurologic disorder, with reported in-hospital mortality rates of 6% to 18% and cognitive or physical sequelae in a large proportion of survivors.^[2–4,26]^ In addition to late institution of appropriate antiviral or immune therapy, several potentially modifiable factors associated with poor outcomes have recently been identified, including delayed ICU admission, cerebral edema on neuroimaging, CSF inflammation, thrombocytopenia, and SE.^[2–4]^ Compared to the other patients in our cohort, patients with early-onset RSE had a higher mortality rate and very poor functional recovery at 3 months. Moreover, RSE present at the onset of encephalitis was an independent risk factor for day-90 mortality. By contrast, early-onset NRSE was not associated with poorer neurologic outcome or mortality. Increasing use of continuous EEG in ICUs has revealed that subclinical seizures are common in patients with acute brain injury.^[24]^ Subclinical seizures are associated with worse outcomes, but it remains to be shown whether their prompt detection and treatment improves the prognosis. Moreover, the potential benefits of routine primary or secondary seizure prophylaxis have yet to be investigated. In the meantime, our findings suggest that early seizure detection and appropriate antiepileptic treatment at the onset of encephalitis may avoid the onset of (super-) refractory SE, a condition associated with very poor outcomes.^[11,12,27]^

This study has the limitations inherent in its retrospective design and lacks external validity. Although our management of patients with acute encephalitis is in line with published guidelines,^[1,28]^ we cannot exclude that our practice differed from other centers, hampering generalization of the study results. Immune-mediated causes of encephalitis, including NMDAR encephalitis, were not systematically sought in the earlier years of this study, and may have been underestimated. The proportion of patients with a probable or definite diagnosis of *Mycobacterium tuberculosis* encephalitis was also unusually high. Tuberculous CNS infections may cause a predominant meningitic syndrome and their clinical course may differ significantly from that of encephalitis due to other causes. However, we have previously reported that these patients had similar clinical presentations at admission to those of the other patients in the cohort.^[4]^ Although we did not find any association between year of admission and outcome, it is likely that prognosis of patients improved significantly over the study period. Compared to patients hospitalized between 1991 and 2001, patients hospitalized after 2001 tended to be older and more frequently immunocompromised, with more severe presentation on ICU admission.^[4]^ We observed significant changes in diagnostic procedures, including an increase in the use of brain magnetic imaging resonance at admission and an increase in the proportion of encephalitis recognized to be of immune-mediated cause. The rate of early onset nonconvulsive seizures at admission may have been underestimated, as EEG monitoring was not systematically performed according to a standardized protocol. Finally, our study may have limited power to detect important predictors of outcome, including older age and immunodepression.

We conclude that coma, cortical involvement on neuroimaging, and nonneurologic organ failure(s) are independent risk factors for SE in patients with acute encephalitis. Conversely, a bacterial etiology is associated with a lower risk of SE. These findings may help identify patients who may benefit from prophylactic AEDs.

## Supplementary Material

Supplemental Digital Content
