# Supplementary material for: Early-onset status epilepticus in patients with acute encephalitis
Source: Medicine (Baltimore). 2016 Jul 29;95(30):e4092. doi: 10.1097/MD.0000000000004092 (PMC5265819; doi:10.1097/MD.0000000000004092)
Supplement: Supplemental Digital Content [file medi-95-e4092-s001.docx]

e-figure1


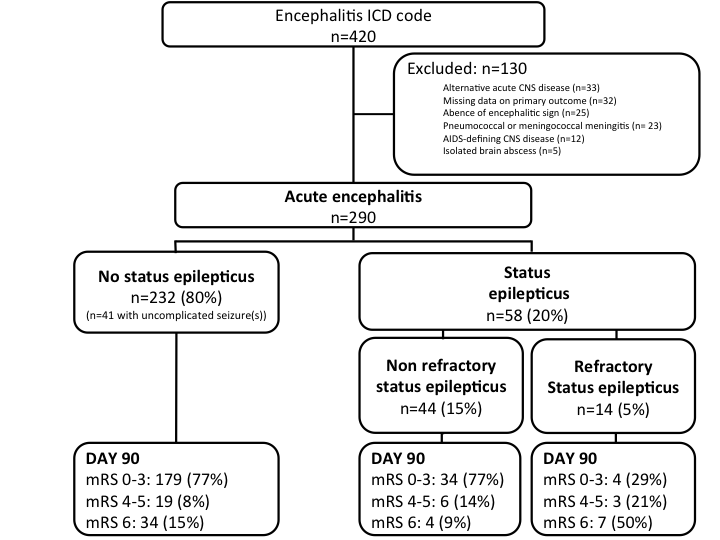


**e-table 1: prehospital use of antiepileptic drugs**

|  | **Missing Data** | **All patients** | **No Status epilepticus** | **Status epilepticus** |  |
| --- | --- | --- | --- | --- | --- |
|  | **n** | **n=290** | **n=232** | **n=58** | ***p*** |
| Prehospital i.v. clonazepam | 0 | 6 (2.0) | 2 (1) | 4 (7) | <0.01 |
| Prehospital 2^nd^ line antiepileptic drug | 0 | 2 (1) | 0 (0) | 2 (3) | <0.01 |
| Prehospital sedation* | 0 | 6 (2) | 2 (1) | 4 (7) | <0.01 |
| Pre-ICU i.v. clonazepam | 2 | 31 (11) | 9 (4) | 22 (38) | <0.01 |
| Pre-ICU 2^nd^ line antiepileptic drug | 0 | 9 (3) | 3 (1) | 6 (10) | <0.01 |
| Pre-ICU sedation | 0 | 32 (11) | 20 (9) | 12 (21) | <0.01 |
| Sedation* on ICU admission | 0 | 138 (48) | 93 (40) | 45 (78) | <0.01 |

*continuous infusion of midazolam and/ or propofol

**e-table 2: characteristics of patients with status epilepticus**

|  |  | **Status epilepticus** | |  |
| --- | --- | --- | --- | --- |
|  | **Missing Data** | **Non refractory** | **Refractory** |  |
|  | **n** | **n=44** | **n=14** | ***p*** |
| Age, years | 0 | 39 (31-54) | 39 (28-64) | 0.84 |
| Male sex | 0 | 25 (57) | 5 (36) | 0.17 |
| Immunocompromised | 0 | 6 (14) | 5 (36) | 0.07 |
| Poor functional status | 0 | 1 (2) | 3 (21) | 0.01 |
| Charlson score | 0 | 0 (0-0) | 0 (0-6) | 0.13 |
| Time between first hospital consultation and ICU admission, days | 0 | 2 (1-5) | 2 (1-12) | 0.54 |
| Pre ICU seizures | 0 | 36 (82) | 14 (100) | 0.09 |
| GCS score | 0 | 8 (6-11) | 8 (6-9) | 0.34 |
| GCS score < 8 | 0 | 19 (43) | 7 (50) | 0.66 |
| Focal sign(s) | 0 | 11 (25) | 3 (21) | 0.79 |
| Temperature, °C | 0 | 38.5 (37.2-39.5) | 38.0 (37.0-38.5) | 0.42 |
| SAPS2 score | 0 | 38 (25-47) | 44 (38-52) | 0.09 |
| Aspiration pneumonia | 0 | 4 (9) | 4 (29) | 0.07 |
| Invasive mechanical ventilation | 0 | 39 (89) | 14 (100) | 0.19 |
| Blood sodium level, mmol/l | 6 | 138 (134-140) | 137 (131-140) | 0.93 |
| Blood sodium level < 130 mmol/l | 6 | 5 (13) | 2 (15) | 0.81 |
| Cerebrospinal fluid |  |  |  |  |
| Cells/microL | 0 | 67 (26-167) | 48 (20-60) | 0.25 |
| Protein level ≥ 1 g/l | 0 | 22 (50) | 5 (36) | 0.35 |
| Abnormal neuroimaging findings, n/total (%) | 0 | 27 (61) | 11 (79) | 0.47 |
| Etiology |  |  |  | 0.79 |
| Infectious | 0 | 19 (43) | 7 (50) |  |
| Immune-mediated | 0 | 8 (18) | 3 (21) |  |
| Undetermined | 0 | 17 (39) | 4 (29) |  |
| Duration of MV, days | 0 | 8 (4-20) | 19 (6-34) | 0.27 |
| Duration of ICU stay, days | 0 | 13 (6-22) | 29 (8-44) | 0.17 |
| Day-90 mortality | 0 | 4 (9) | 7 (50) | <0.01 |
| Day-90 Rankin 4-6 | 0 | 10 (23) | 10 (71) | <0.01 |
